# Poor long-term outcomes of intravenous drug users with infectious endocarditis

**DOI:** 10.1016/j.xjon.2022.05.013

**Published:** 2022-05-31

**Authors:** Juan Caceres, Aroosa Malik, Tom Ren, Aroma Naeem, Jeffrey Clemence, Alexander Makkinejad, Xiaoting Wu, Bo Yang

**Affiliations:** a University of Michigan Medical School, Ann Arbor, Mich; b Department of Cardiac Surgery, Michigan Medicine, Ann Arbor, Mich

**Keywords:** cardiac surgery, endocarditis, outcomes, aortic valve surgery, tricuspid valve surgery, CHF, congestive heart failure, HR, hazard ratio, IVDU, intravenous drug use, MET, multidisciplinary endocarditis team

## Abstract

**Objectives:**

The optimal management of active endocarditis in intravenous (IV) drug users is still lacking.

**Methods:**

From the years 1997 to 2017, 536 patients with active infectious endocarditis were surgically treated, including 83 (15%) with IV drug use (IVDU) and 453 (85%) without IV drug use (non-IVDU). Initial data were obtained from the Society of Thoracic Surgeons database and supplemented with chart review and national death index data.

**Results:**

The IVDU group was significantly younger (43 vs 56 years old) than the non-IVDU group and had greater rates of psychiatric disorders, drug use, and tricuspid valve endocarditis (28% vs 8.6%). Hypertension, dyslipidemia, and diabetes mellitus were significantly more common in the non-IVDU group. Perioperative complications and operative mortality (7.2% vs 7.9%) were similar. IVDU was not a significant risk factor for operative mortality. Kaplan–Meier survival was significantly lower in the IVDU group (5-year survival, 46% vs 67%). Significant risk factors for long-time mortality included IV drug use (hazard ratio [HR], 1.92), age ≥65 years (HR, 1.78), congestive heart failure (HR, 1.87), and enterococcus endocarditis (HR, 1.54). The 5-year rate of reoperation was similar between IVDU and non-IVDU groups (2.4% vs 2.7%).

**Conclusions:**

IVDU is a significant risk factor for long-term mortality. A multidisciplinary approach was preferred for IVDU patients to treat both endocarditis and substance use disorder and improve long-term survival.

## Graphical abstract

**Figure:**
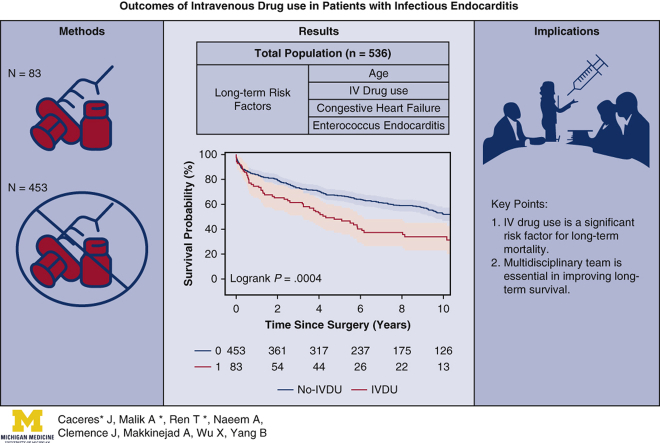

**Figure:**
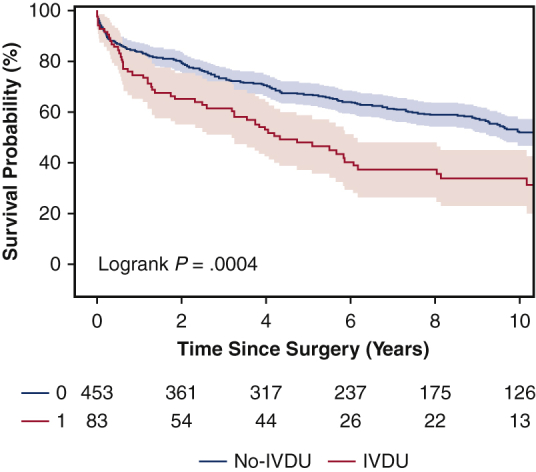
Survival of IVDU and non-IVDU patients after surgery.

**Central Message**

Endocarditis in IVDU requires multidisciplinary treatment. Surgery alone is not sufficient.

**Perspective**

Although IV drug usage as the etiology of endocarditis did not increase the incidence of short-term complications in patients with active infectious endocarditis compared with non-IVDU, the long-term survival was very poor due to addiction as a second disease. Surgery alone may not be adequate for this patient population.

Active infective endocarditis is a disease process associated with increased morbidity and mortality. Of patients with acute endocarditis, 25% to 30% require surgical intervention, with a noted surgical mortality of 8% to 16%.^1^^,^^2^ The incidence of infectious endocarditis has been on the rise, increasing from 11 to 15 cases per 100,000 people between 2000 and 2011.^3^, ^4^, ^5^ This trend has continued despite the decline in rheumatic fever cases, a predominant cause of infectious endocarditis, as well as improvements in health care. An increase in intravenous drug use (IVDU) in the setting of the opioid epidemic is a potential contributing factor to the increase in infectious endocarditis.

The sale of opioid pain relievers increased by 4 times between the years 1999 and 2010, as noted by the Center of Disease Control and Prevention.^6^ Kadri and colleagues^7^ recently reported a significantly increased incidence of infectious endocarditis due to IVDU between 2002 and 2016 with an annual percent chance of 1.8%. Despite this alarming increase, the current literature regarding long-term outcomes for IVDU compared with non-IVDU is conflicting. Furthermore, studies have demonstrated similar survival between the 2 groups whereas others have demonstrated poor long-term outcomes in IVDU.^5^^,^^8^^,^^9^

As such, we examined the short- and long-term outcomes of IVDU and non-IVDU patients who underwent surgical intervention for infectious endocarditis at our institution. We hypothesized that intravenous (IV) drug users have similar short- and long-term outcomes.

## Methods

This study was approved by the institutional review board (HUM0014297; March 31, 2018), and a waiver of informed consent was obtained.

### Patient Selection

Between the years 1997 and 2017, 536 patients with active endocarditis underwent surgical management at our institution. Patients who were treated medically or turned down for surgery were excluded. Endocarditis involvement for these patients included the aortic valve, mitral valve, tricuspid valve, or multiple valves. These patients were further divided based on their history of IVDU. Of the 536 patients, 83 (15%) had a history of IVDU and 453 (85%) were non-IV drug users. Patients in the IVDU group were all IV drug users, but it was difficult to ascertain which patients were actively using IV drugs at the time of their surgery. IVDU was not limited by heroin use—some patients injected cocaine, methamphetamine, or were unspecified drug users.

### Data Collection

Data elements were obtained from the Society of Thoracic Surgeons from our institution's Cardiac Surgery Data Warehouse to determine pre-, intra-, and postoperative characteristics. Investigators supplemented data collection through a review of medical records and operative reports using traditional chart review as well as the Electronic Medical Record Search Engine data-retrieval tool.^10^ Survival data were obtained through the National Death Index database through December 12, 2021, and supplemented with medical record review and questionnaire response via mailed survey or phone call through February 2019.^11^ Reoperation events included reoperation of prosthesis for any reason and for reinfection.

### Statistical Analysis

Data are presented as median (interquartile range) for continuous data and n (%) for categorical data. Univariate comparisons between IVDU and non-IVDU were performed using χ^2^ tests for categorical data. The Kolmogorov–Smirnov test was used to test the normality of the data. Univariate comparisons between groups were performed using the Mann–Whitney *U* test for continuous data. Multivariable logistic regression was used to calculate the odds ratios of risk factors for operative mortality. The risk factors included group, age, sex, cirrhosis, congestive heart failure (CHF), cardiogenic shock, and preoperative sepsis. Cox proportional hazard regression was performed to calculate the hazard ratio (HR) for late mortality including group, age ≥65 years, sex, CHF, enterococcus endocarditis, cirrhosis, and preoperative sepsis. The C-statistic for the whole population model was 0.64. All variables passed the proportional hazards assumption for this model. For the proportional hazards regression model within IV drug uses, the C-statistic was 0.66. The *Enterococcus* variable was used as a strata variable as it did not pass the proportional hazards assumption. A Kaplan–Meier survival curve was used to assess long-term survival using time to death since valve operation due to endocarditis. The log-rank test was used to compare survival between groups (IVDU and non-IVDU). Cumulative incidence function curves for reoperation were generated adjusting for the competing risk of death. The Gray test was used to assess the difference in the cumulative incidence function curves between groups. Statistical calculations were performed using SAS (SAS Institute), SPSS, Version 28 (IBM Corp), and Quickcalcs (GraphPad).

## Results

### Demographics, Comorbidities, Substance Use, and Psychiatric Disorders

Patients in the IVDU group were significantly younger than patients in the non-IVDU group (43 vs 56 years) and had significantly greater rates of psychiatric disorders, including anxiety (18% vs 6.6%; *P* < .001) and bipolar disorder (7.2% vs 2.2%; *P* = .03). There were also significantly greater rates of tobacco use (53% vs 16%; *P* < .001), heroin use (47% vs 0%; *P* < .001), cocaine use (31% vs 1.3%; *P* < .001), methamphetamine use (4.8% vs 0.2%; *P* = .002), pain medication use (18% vs 0.4%; *P* < .001), alcohol abuse (17% vs 8.4%; *P* = .02), and other unspecified drug use. In contrast, the non-IVDU group had significantly greater rates of diabetes, dyslipidemia, and hypertension. Other preexisting comorbidities such as coronary artery disease, arrhythmia, cirrhosis, and CHF were not significantly different between the 2 groups (Table 1).

**Table 1:** Demographics, comorbidities, substance use, and psychiatric disorders

Variable	Non-IVDU (n = 453)	IVDU (n = 83)	*P* value
Age, y (median)	56 (44, 65)	43 (33, 48)	**<.001**
Sex (female)	130 (29)	30 (36)	.17
Comorbidities			
Coronary artery disease	94 (21)	15 (18)	.58
Diabetes	117 (26)	10 (12)	**.007**
Dyslipidemia	171 (38)	10 (12)	**<.001**
Hypertension	263 (58)	36 (43)	**.013**
Cirrhosis	17 (3.8)	7 (8.4)	.09
MELD 0-9	5 (1.1)	3 (3.6)	.11
MELD 10-19	11 (2.4)	3 (3.6)	.46
MELD 20-29	1 (0.2)	1 (1.2)	.29
Congestive heart failure	229 (51)	46 (55)	.41
Stroke	88 (19)	15 (18)	.77
Arrhythmia	67 (15)	7 (8.4)	.12
Moderate-to-severe lung disease	37 (8.2)	3 (3.6)	.15
Psychiatric disorders			
Depression	51 (11)	13 (16)	.26
Anxiety	30 (6.6)	15 (18)	**<.001**
Bipolar disorder	10 (2.2)	6 (7.2)	**.03**
History of substance use			
Tobacco use			
Nonsmoker	255 (56)	18 (22)	**<.001**
Former smoker	128 (28)	21 (25)	.58
Current smoker	70 (16)	44 (53)	**<.001**
Alcohol abuse	38 (8.4)	14 (17)	**.02**
Heroin∗	0 (0)	39 (47)	**<.001**
Cocaine	6 (1.3)	26 (31)	**<.001**
Pain medication	2 (0.4)	15 (18)	**<.001**
Bath salts	0 (0)	1 (1.2)	.16
Benzodiazepine	0 (0)	1 (1.2)	.16
Methamphetamine	1 (0.2)	4 (4.8)	**.002**
Unspecified polysubstance drug use	2 (0.4)	5 (6.0)	**.001**
Unspecified drug use	3 (0.7)	25 (30)	**<.001**
Other	10 (2.2)	4 (4.8)	.25

Data presented as median (25%, 75%) for continuous data and n (%) for categorical data. The variables in bold are statistically significant. *Non*-*IVDU*, No intravenous drug use; *IVDU*, intravenous drug use; *MELD*, Model for End-Stage Liver Disease.

∗ IV drug use was not limited by heroin use. Some patients injected cocaine, methamphetamine, or were unspecified drug users.

### Preoperative Data

Patients with IVDU were significantly more likely to have tricuspid valve endocarditis (28% vs 8.6%; *P* < .001) and a history of endocarditis (24% vs 13%; *P* = .007). The IVDU group had greater rates of endocarditis due to *Staphylococcus aureus* (34% vs 23%; *P* = .04), and fungi (9.6% vs 2.2%; *P* = .003). Preoperatively, the IVDU group had significantly greater rates of embolic events to the lungs (24% vs 6.2%; *P* < .0001), spleen (13% vs 6.2%; *P* = .02), kidneys (12% vs 3.1%; *P* = .001), and extremities (13% vs 5.1%; *P* = .005). There were no significant differences between the groups regarding aortic insufficiency, root abscess, root aneurysm, cardiogenic shock, or wait times in the setting of active endocarditis (Table 2).

**Table 2:** Preoperative data

Variable	Non-IVDU (n = 453)	IVDU (n = 83)	*P* value
Endocarditis type			
Aortic valve	282 (62)	43 (52)	.73
Mitral valve	200 (44)	36 (43)	.90
Tricuspid valve	39 (8.6)	23 (28)	**<.001**
Pulmonic valve	4 (0.9)	0 (0)	1.0
Causative microorganism			
Staphylococci	170 (38)	31 (37)	.23
*Staphylococcus aureus*	105 (23)	28 (34)	**.04**
Coagulase-negative staphylococci	65 (14)	3 (3.6)	**.007**
Enterococci	65 (14)	13 (16)	.76
Streptococci	115 (25)	15 (18)	.15
Gram-negative rods	17 (3.8)	4 (4.8)	.55
Fungal	10 (2.2)	8 (9.6)	**.003**
Multiple	3 (0.7)	3 (3.6)	**.5**
Others∗	10 (2.2)	2 (2.4)	1.0
Culture negative	63 (14)	7 (8.4)	.17
History of endocarditis	58 (13)	20 (24)	**.007**
Cardiogenic shock	30 (6.6)	8 (9.6)	.33
Pneumonia	44 (9.7)	8 (9.6)	.98
Sepsis	89 (20)	23 (28)	.10
Aortic insufficiency			
Moderate	49 (11)	6 (7.2)	.32
Severe	142 (31)	26 (31)	1.0
Aortic stenosis	150 (33)	26 (31)	.75
Root abscess	157 (35)	22 (27)	.58
Preoperative embolic events	189 (42)	54 (65)	
Brain/CNS	104 (23)	21 (25)	.64
Lungs	28 (6.2)	20 (24)	**<.001**
Spleen	28 (6.2)	11 (13)	**.02**
Kidneys	14 (3.1)	10 (12)	**.001**
Eyes	5 (1.1)	1 (1.2)	1.0
Extremities	23 (5.1)	11 (13)	**.005**
Liver	3 (0.7)	2 (2.4)	.17
Heart	10 (2.2)	2 (2.4)	1.0
Other	28 (6.2)	7 (8.4)	.45
Previous cardiac surgery∗	154 (34)	19 (23)	
CABG	49 (11)	2 (2.4)	**.16**
Aortic valve repair/replacement	113 (25)	12 (15)	**.04**
Mitral valve repair/replacement	41 (9.1)	7 (8.4)	.81
Tricuspid valve repair/replacement	6 (1.3)	1 (1.2)	1.0
Pulmonary valve repair/replacement	4 (0.9)	0 (0)	1.0
Ascending or arch repair/replacement	15 (3.3)	0 (0)	.14
Incidence of cardiovascular surgery			
First surgery	284 (63)	63 (76)	**.02**
First reoperation	127 (28)	17 (21)	.18
Second or more reoperations	42 (9.3)	3 (3.6)	.88
Wait time, d	6 (3, 12)	7 (3, 12)	.76

Data presented as median (25%, 75%) for continuous data and n (%) for categorical data. The variables in bold are statistically significant. *Non*-*IVDU*, No intravenous drug use; *IVDU*, intravenous drug use; *CNS*, central nervous system; *CABG*, coronary artery bypass grafting.

∗ Not including valve valvotomy or valvuloplasty.

### Operative Data

The IVDU group had significantly greater rates of tricuspid valve repair (25% vs 15%; *P* = .02) and replacement (16% vs 3.1%, *P* < .001) but similar rates of other valve procedures compared with the non-IVDU group. The IVDU group had significantly fewer ascending aorta and aortic root procedures but similar rates of coronary artery bypass grafting, hemiarch, and total arch procedures. Cardiopulmonary bypass and crossclamp times were also similar between groups (Table 3).

**Table 3:** Operative data

Variable	Non-IVDU (n = 453)	IVDU (n = 83)	*P* value
Status			
Elective	34 (7.5)	2 (2.4)	.09
Urgent	368 (81)	70 (84)	.50
Emergent	51 (11)	11 (13)	.60
Circulatory arrest	27 (6.0)	3 (3.6)	.60
CPB time, min	171 (117, 254)	142 (95, 231)	.15
Crossclamp time, min	135 (87, 203)	106 (70, 175)	.07
Aortic valve procedure			
Repair	7 (1.5)	0 (0)	.60
Replacement	282 (62)	44 (53)	.11
Implantation technique∗			
Total	23 (5.1)	4 (4.8)	1.0
Modified inclusion	124 (27)	15 (18)	.08
Subcoronary	7 (1.5)	0 (0)	.60
Mitral valve procedure			
Repair†	132 (29)	25 (30)	.86
Replacement	103 (23)	21 (25)	.67
Tricuspid valve procedure			
Repair	68 (15)	21 (25)	**.02**
Replacement	14 (3.1)	13 (16)	**<.001**
Pulmonic valve procedure			
Repair	1 (0.2)	1 (1.2)	.29
Replacement	2 (0.4)	0 (0)	1.0
Concomitant procedures			
CABG	46 (10)	6 (7.2)	.41
Aortic root procedure	190 (42)	23 (28)	**.02**
Ascending aorta procedure	57 (13)	3 (3.6)	**.02**
Aortic hemiarch procedure	6 (1.3)	0 (0)	.60
Total aortic arch procedure	2 (0.4)	0 (0)	1.0

Data presented as median (25%, 75%) for continuous data and n (%) for categorical data. The variables in bold are statistically significant. *Non*-*IVDU*, No intravenous drug use; *IVDU*, intravenous drug use; *CPB*, cardiopulmonary bypass; *CABG*, coronary artery bypass grafting.

∗ Implantation technique for stentless valves.

† Repair includes repair, reconstruction, or annuloplasty.

### Perioperative Outcomes

IVDU patients had a lower rate of postoperative atrial fibrillation (17% vs 27%; *P* = .04) and a greater rate of postoperative pacemakers (12% vs 5.5%; *P* = .03) compared with non-IVDU patients. Nevertheless, there were no significant differences in other perioperative complications or mortality (operative mortality: 7.2% vs 7.9%) between groups. ICU stays, ventilation hours, and red blood cell units given were also similar between IVDU and non-IVDU groups (Table 4). IVDU was not a significant risk factor for operative mortality with an odds ratio of 1.02, *P* = .97 (Table 5).

**Table 4:** Postoperative outcomes

Variable	Non-IVDU (n = 453)	IVDU (n = 83)	*P* value
RBC units	1 (0.0, 4.0)	1 (0.0, 4.0)	.28
Ventilation hours	7.4 (0.0, 33)	6.0 (1.5, 28)	.99
ICU stay, d	2.1 (0.0, 6.6)	2.0 (0.0, 4.3)	.41
Reoperation for bleeding	12 (2.6)	2 (2.4)	1.0
Planned delayed sternal closure	5 (1.1)	2 (2.4)	.30
Sternal dehiscence	2 (0.4)	0 (0)	1.0
Sepsis	11 (2.4)	1 (1.2)	.70
Positive blood cultures	12 (2.6)	2 (2.4)	1.0
Stroke	8 (1.8)	0 (0)	.62
Paralysis	2 (0.4)	0 (0)	1.0
Prolonged ventilation	134 (30)	24 (29)	.90
Pneumonia	36 (7.9)	8 (9.6)	.61
Device			
Pacemaker	25 (5.5)	10 (12)	**.03**
ICD	3 (0.7)	0 (0)	1.0
Pacemaker/ICD	3 (0.7)	0 (0)	1.0
Cardiac arrest	15 (3.3)	1 (1.2)	.49
Multisystem organ failure	8 (1.8)	0 (0)	.62
Gastrointestinal event	37 (8.2)	2 (2.4)	.06
Atrial fibrillation	124 (27)	14 (17)	**.04**
In-hospital mortality	31 (6.8)	6 (7.2)	.90
30-d mortality	32 (7.1)	6 (7.2)	.96
Intraoperative mortality	8 (1.8)	2 (2.4)	.66
Operative mortality∗	36 (7.9)	6 (7.2)	.82

Data presented as median (25%, 75%) for continuous data and n (%) for categorical data. The variables in bold are statistically significant. *Non*-*IVDU*, No intravenous drug use; *IVDU*, intravenous drug use; *RBC*, red blood cell; *ICU*, intensive care unit; *ICD*, implanted cardioverter defibrillator.

∗ Operative mortality: based on the Society of Thoracic Surgeons definition and includes all deaths, regardless of cause, occurring during the hospitalization in which the operation was performed, even if after 30 days (including patients transferred to other acute care facilities); and all deaths, regardless of cause, occurring after discharge from the hospital, but before the thirtieth postoperative day.

**Table 5:** Risk factors for operative mortality

Variable	Odds ratio (95% confidence intervals)	*P* value
IV drug use	1.02 (0.38, 2.71)	.97
Age	1.02 (1.0, 1.04)	.12
Sex (female)	1.38 (0.70, 2.70)	.35
Cirrhosis	1.93 (0.53, 7.0)	.32
CHF	1.62 (0.83, 3.19)	.16
Cardiogenic shock	1.56 (0.55, 4.42)	.40
Preoperative sepsis	1.41 (0.67, 2.95)	.37

*IV*, Intravenous; *CHF*, congestive heart failure.

### Long-Term Outcomes

**Figure 1:**
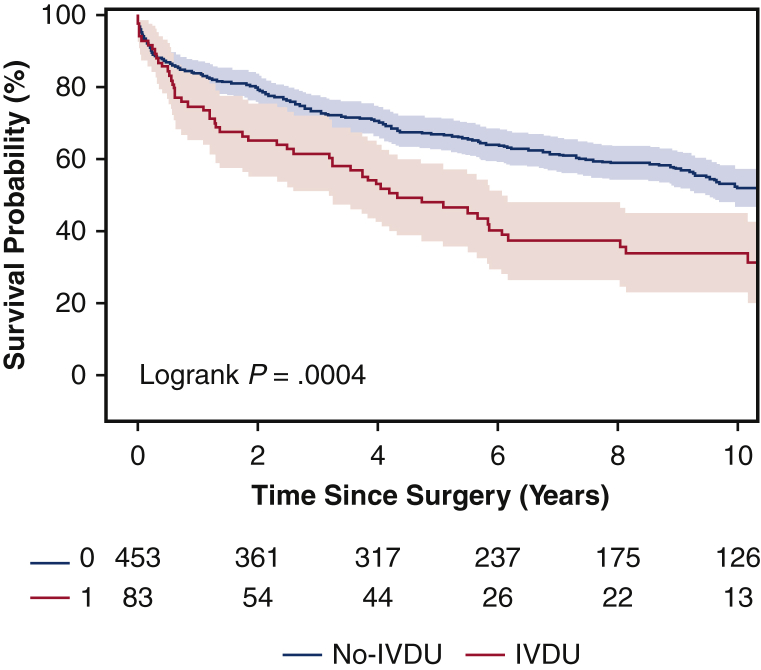
Kaplan–Meier mid-term survival of patients who underwent surgical management for active infectious endocarditis in intravenous drug users (*IVDU*; 5-year: 46%; 95% CI, 36%-57%) and non-IVDU (5-year: 67%; 95% CI, 63%-71%).

**Figure 2:**
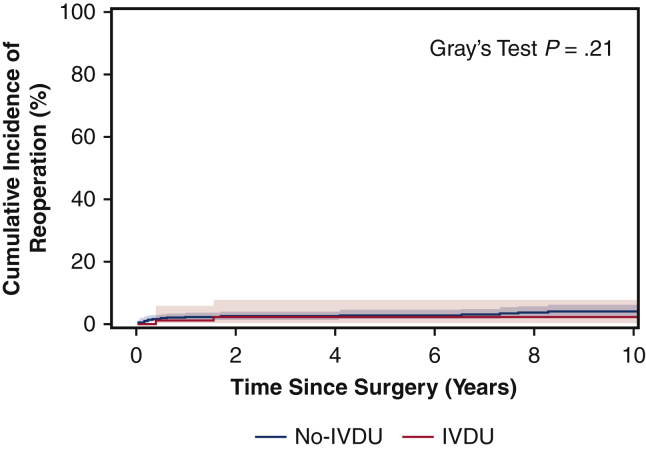
The cumulative incidence for reoperation in intravenous drug users (*IVDU*; 5-year: 2.4%; 95% CI, 0.5%-7.6%) and non-IVDU (5-year: 2.7%; 95% CI, 1.5%-4.5%).

The 5-year survival rates were 46% for the IVDU group, and 67% for the non-IVDU group, respectively (*P* = .0004) (Figure 1). Moreover, IVDU was a significant risk factor for long-term mortality (HR, 1.92) as well as age ≥65 years (HR, 1.78), CHF (HR, 1.87), and *Enterococcus endocarditis* (HR, 1.54) (Table 6). Among IV drug users, age (HR, 1.04), female sex (HR, 2.17), and CHF (HR, 2.40) were significant risk factors for long-term mortality (Table 7). The 5-year postoperative cumulative incidence of reoperation was similar between IVDU and non-IVDU groups using death as a competing factor (2.4% vs 2.7%) (Figure 2).

**Table 6:** Risk factors for long-term mortality

Variable	Hazard ratio (95% confidence intervals)	*P* value
IV drug use	1.92 (1.39, 2.64)	**<.0001**
Age ≥65 y	1.78 (1.34, 2.35)	**<.0001**
Sex (female)	1.24 (0.96 1.60)	.10
CHF	1.87 (1.46, 2.39)	**<.0001**
*Enterococcus*	1.54 (1.12, 2.10)	**.007**
Cirrhosis	1.32 (0.75, 2.34)	.34
Preoperative sepsis	1.06 (0.78, 1.44)	.71

The variables in bold are statistically significant. *IV*, Intravenous; *CHF*, congestive heart failure.

**Table 7:** Risk factors for long-term mortality among IV drug users∗

Variable	Hazard ratio (95% confidence intervals)	*P* value
Age	1.04 (1.02, 1.10)	**.04**
Sex (female)	2.17 (0.92, 5.58)	**.03**
CHF	2.40 (0.88, 3.58)	**.01**
Cirrhosis	0.93 (1.13, 6.22)	.87
Preoperative Sepsis	0.57 (0.26, 1.70)	.18

The variables in bold are statistically significant. *CHF*, Congestive heart failure.

∗ *Enterococcus* as a causative organism was set as a strata for model.

## Discussion

In this study, we found that IVDU did not significantly increase short-term complications or operative mortality in patients with infective endocarditis. The greater rate of postoperative atrial fibrillation in the non-IVDU group was likely related to the older age of the patients and the greater rates of comorbidities in this group. The cumulative incidence of reoperation for endocarditis was also similar between groups. Nevertheless, there was significantly lower long-term survival associated with IVDU on Kapan Meier analysis (Figure 1). IVDU (HR, 1.92) was a significant risk factor for long-term mortality. Additional risk factors for late mortality included age ≥65 years, CHF, and *E endocarditis*.

The IVDU group had significantly more psychiatric disorders and preoperative embolic events (Tables 1 and 2). IV drug users were more likely to have anxiety, bipolar disorder, as well as additional substance use including alcohol, heroin, cocaine, pain medications, methamphetamines, or other drugs. A meta-analysis performed by Conner and colleagues^12^ demonstrated a positive association between intravenous drug use and depression, supporting the idea that IV drug abuse may be a complication of a patient's psychiatric conditions. Given the IV access for drug use in these patients, it was expected for IVDU patients to have more tricuspid valve endocarditis as shown in our study (Table 2) and other studies.^13^ If a physician encounters a patient with tricuspid valve endocarditis and a history of psychiatric diseases, IVDU should be suspected. At our institution, for all patients with endocarditis, if the valve was salvageable, it was repaired. Throughout the 21-year experience, more mitral and tricuspid valves were repaired than aortic valves. When patch repair of the valve or aortic root was required, autologous pericardium was preferred to minimize prosthetic material or bovine pericardium. If the valve required replacement, a bioprosthetic valve or a mechanical valve was used for patients. For tricuspid valve, we did not use tricuspid valvectomy for those patients. We either repaired or replaced the tricuspid valve.

With regards to perioperative outcomes, our study did not show any significant difference in operative mortality between the IVDU and non-IVDU groups. This is in concordance with a large meta-analysis, which did not show any significant difference in all-cause perioperative mortality between IVDU and non-IDVU groups.^14^ A large retrospective study with more than 34,000 patients showed lower unadjusted operative mortality among IV drug users, with greater risk of major morbidity after adjusting for other factors.^15^ Nevertheless, the adjusted odds ratio for operative mortality was not significant. The operative mortality in this study was 10.7% in the non-IVDU group compared with 6.7% in the IVDU group. Although our sample size was smaller, our operative mortality was similar (7.9% vs 7.2%). It is likely that the lack of difference in perioperative mortality between groups is because IV drug users were significantly younger and had fewer comorbidities than their non-IVDU counterparts.

Consistent with multiple studies, the long-term survival of our patients after surgical intervention was much worse in IVDU patients compared with non-IVDU patients.^5^^,^^9^^,^^16^ One-half of the IVDU patients died 4 years after surgery (Figure 1). Based on the entire cohort, risk factors for long-term mortality in our study were IVDU, age ≥65 years, CHF, and *E endocarditis* (Table 6). We should therefore be cautious to offer surgery to older patients and patients with active infectious endocarditis and CHF. *E endocarditis* should also be treated aggressively, as it has been shown to have the greatest 1-year mortality along with *Staphylococcus aureus* according to a study by Shah and colleagues.^17^

Among IV drug users with infective endocarditis, we should also be prepared to offer holistic care. A recent study found that the leading cause of death for IVDU patients with endocarditis after surgery was recidivism compared with malignancy, cardiopulmonary, and kidney disease for the non-IVDU.^5^ As surgeons, we can treat endocarditis in IV drug users with fairly good perioperative results comparable with non-IV drug users. However, if we just operated on those patients and left the psychiatric disorder and substance use disorder untreated, we would not be treating these patients adequately.

Interestingly, among these IV drug users, long-term survivors were younger while long-term nonsurvivors of IVDU patients had significantly greater rates of CHF (Tables E1 and E2). There were more triscuspid valve replacements in survivors, but post-operative outcomes were similar between survivors and non-survivors (Tables E3 and E4). The significant risk factors for late death within the IVDU group included age, female sex, and CHF, suggesting that we should be cautious in offering surgery to older female IVDU patients, especially if they have CHF (Table 7).

In 2018, the Multidisciplinary Endocarditis Team (MET), a collaborative environment for the discussion and management of every endocarditis case, was established at our institution. A multidisciplinary approach was used to treat patients in the inpatient and outpatient setting with the help of social workers, pharmacists, and physicians from infectious disease, cardiology, cardiac surgery, and neurology. Indications for surgery included but were not limited to (1) valve dysfunction resulting in heart failure; (2) left-sided endocarditis caused by highly resistant microorganisms; (3) the presence of heart block, root abscess, or destructive lesions; (4) persistent or fever despite multiple days of appropriate antibiotics; (5) and large or persistent vegetations.^18^^,^^19^

In terms of structure, patients were typically discussed before surgery, but also discussed again if any problems arose. Postoperatively, patients were in the cardiovascular intensive care unit until they were stabilized and then transferred to a step-down medicine service. The patients were then followed by cardiac surgery and any of the other medical specialties as needed. This change led to a substantial impact on the management (diagnosis, treatment, communication, mortality) of all endocarditis patients as evidenced by a significant decrease in in-hospital mortality from 29.4% to 7.1% between 2014 and 2019 at the University of Michigan, although patients in hospice were not included.^20^^,^^21^ Furthermore, Chirillo and colleagues^22^ reported a 2-fold decrease in in-hospital mortality after implementation of their multidisciplinary protocol.

Given the complexity of many of these patients, multidisciplinary teams are essential for successful management of the short- and long-term survival of these patients both in the in-patient and out-patient settings. This is particularly important in patients with IVDU, who have greater rates of readmission and disease recurrence.^23^ Although psychiatry and addiction medicine are not part of our core multidisciplinary endocarditis conference attendance, our colleagues recommend attendance of these teams as part of treatment in patients with substance abuse disorder, complicating the diagnosis of endocarditis as in the case of many IVDU patients.^24^ We have both of these teams at our institution, and it is the responsibility of the primary care team to make the appropriate consults if necessary. As part of the multidisciplinary endocarditis team, it would be beneficial to make this part of the recommendations as indicated for these patients. There should also be a movement to enhance training in addiction within infectious disease as to provide an additional perspective in the multidisciplinary conference when a formal addiction team is not available.^25^

The cumulative incidence of reoperation was overall similar between the groups (Figure 2). The relatively low reoperation rate supported our choice of bioprosthesis for patients who needed a valve replacement. Kaiser and colleagues^8^ noted greater rates of reoperation in IV drug users as compared with non-IV drug users, likely from reinfection. However, the reoperation rates between the IVDU group and the non-IVDU group were comparable in our study, which was likely obscured by the high mortality of the IVDU group. By operating on patients with recurrent endocarditis due to relapse of IVDU, we are not targeting the underlying cause and contributing to additional morbidity with each reoperation for the same disease. Therefore, before operating on these patients, we emphasize that our group does not perform any additional surgeries related to relapse from IVDU. This could also be a contributor to the low reoperation rate.

Nevertheless, if patients developed prosthetic valve endocarditis without relapse of IVDU, we performed a second operation. If they developed prosthetic valve endocarditis due to relapse of IVDU, most surgeons did not perform a reoperation, especially in patients without good social support. We also discuss these cases with the MET. In such cases, it would be beneficial to include the medical ethics team in this discussion, which is available at our institution. Our MET's recommendations have led to surgery when an operation was not planned, indicating that these discussions can change management. Additionally, we now focus on providing patients with multidisciplinary care to prevent recidivism and therefore reinfection.

In summary, surgical intervention alone for IVDU patients is not enough. More needs to be done to combat the psychosocial comorbidities that exist in these patients. With surgery, we help alleviate the active infectious process and prevent poor short-term outcomes. However, in the long-run, proper treatment of underlying psychiatric disorders, and substance use is key for successfully decreasing morbidity and mortality in patients with IVDU. Furthermore, a standardized multidisciplinary approach may also be beneficial.^20^^,^^24^^,^^26^ As such, guidelines highlighting surgical management in IV drug users for first-time and recurrent disease, including recommendations for substance use interventions and multidisciplinary team-based care, should continue to be developed (Video 1).

This study is a single-center experience and is therefore limited in breadth. It also has all the limitations of a retrospective study. We had a large sample of patients with endocarditis but had a relatively small sample size of patients with IVDU. Our survey response rate was not 100% and could have underestimated the cumulative incidence of reoperation. The study was also limited by the possibility of Type II error. Nevertheless, our Cox model was able to provide us with enough sensitivity to show the considerable effect of IVDU on long-term mortality in patients with endocarditis.

## Conclusions

**Figure 3:**
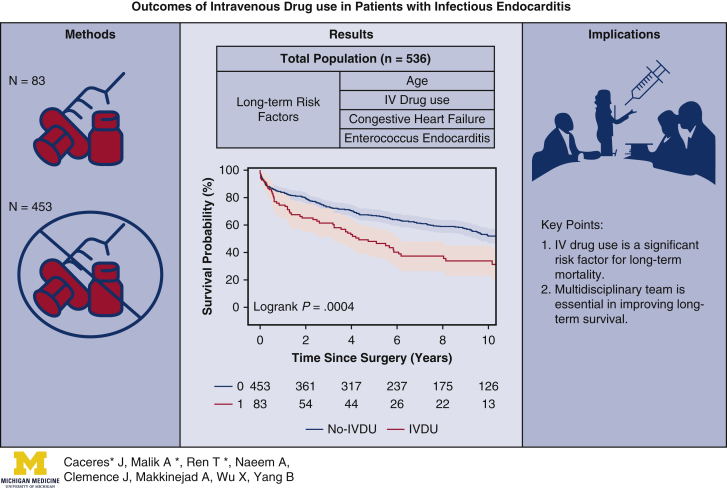
IV drug use is a significant risk factor for long-term mortality. A multidisciplinary approach is essential for patients with IV drug use to treat both endocarditis and substance use disorder and improve long-term survival. *IV*, Intravenous; *IVDU*, intravenous drug users.

Surgery should be cautiously offered to IVDU patients, who have a significantly greater risk for long-term mortality. A multidisciplinary approach was preferred for IVDU patients to treat both endocarditis and substance use disorder and improve long-term survival (Figure 3).

### Conflict of Interest Statement

The authors reported no conflicts of interest.

The *Journal* policy requires editors and reviewers to disclose conflicts of interest and to decline handling or reviewing manuscripts for which they may have a conflict of interest. The editors and reviewers of this article have no conflicts of interest.

## Figures and Tables

**Table E1:** Demographics, comorbidities, substance use, and psychiatric disorders in IV drug users

Variable	Survivor (n = 29)	Nonsurvivor (n = 54)	*P* value
Age, y (median)	37 (26, 44)	45 (38, 50)	**.001**
Sex (female)	10 (35)	20 (37)	.53
Comorbidities			
Coronary artery disease	2 (6.9)	13 (24)	.05
Diabetes	2 (6.9)	8 (15)	.29
Dyslipidemia	6 (21)	4 (7.4)	.16
Hypertension	11 (38)	25 (46)	.46
Cirrhosis	1 (3.4)	6 (11)	.43
MELD 0-9	1 (3.4)	2 (3.7)	1.0
MELD 10-19	0 (0)	3 (5.6)	.50
MELD 20-29	0 (0)	1 (1.9)	1.0
Congestive heart failure	9 (31)	37 (69)	**.001**
Stroke	6 (21)	9 (17)	.65
Arrhythmia	3 (10)	4 (7.4)	.96
Moderate-to-severe lung disease	1 (3.4)	2 (3.7)	1.0
Psychiatric disorders			
Depression	4 (14)	9 (17)	.98
Anxiety	5 (17)	10 (19)	.89
Bipolar disorder	2 (6.9)	4 (7.4)	1.0
History of substance use			
Tobacco use			
Nonsmoker	5 (17)	13 (24)	.47
Former smoker	7 (24)	14 (26)	.86
Current smoker	17 (59)	27 (50)	.45
Alcohol abuse	1 (3.4)	13 (24)	.04
Heroin∗	15 (52)	24 (44)	.40
Cocaine	7 (24)	19 (35)	.30
Pain medication	5 (17)	10 (19)	.89
Bath salts	1 (3.4)	0 (0)	.75
Benzodiazepines	0 (0)	1 (1.9)	1.0
Methamphetamines	0 (0)	4 (7.4)	.34
Unspecified polysubstance drug use	2 (6.9)	3 (5.6)	1.0
Unspecified drug use	8 (28)	17 (32)	.71
Other	1 (3.4)	3 (5.6)	1.0

Data presented as median (25%, 75%) for continuous data and n (%) for categorical data. The variables in bold are statistically significant. *MELD*, Model for End-Stage Liver Disease.

∗ IV drug use was not limited by heroin use. Some patients injected cocaine, methamphetamine, or were unspecified drug users.

**Table E2:** Preoperative data in IV drug users

Variable	Survivor (n = 29)	Nonsurvivor (n = 54)		*P* value
Endocarditis type				
Aortic valve	12 (41)		31 (57)	.16
Mitral valve	11 (38)		25 (46)	.46
Tricuspid valve	12 (41)		11 (20)	**.04**
Pulmonic valve	0 (0)		0 (0)	1.0
Causative microorganism				
Staphylococci	14 (48)		17 (31)	.16
*Staphylococcus aureus*	13 (45)		15 (28)	.12
Coagulase-negative staphylococci	1 (3.4)		2 (3.7)	1.0
Enterococci	2 (6.9)		11 (20)	.20
Streptococci	6 (21)		9 (17)	.65
Gram-negative rods	2 (6.9)		2 (3.7)	.91
Fungal	3 (10)		5 (9.3)	1.0
Multiple	0 (0)		3 (5.6)	.50
Others∗	1 (3.4)		1 (1.9)	1.0
Culture negative	1 (3.4)		6 (11)	.43
History of endocarditis	6 (21)		14 (26)	.60
Cardiogenic shock	3 (10)		5 (9.3)	1.0
Pneumonia	4 (14)		4 (7.4)	.58
Sepsis	14 (48)		9 (17)	**.002**
Aortic insufficiency				
Moderate	2 (6.9)		4 (7.4)	1.0
Severe	4 (14)		22 (41)	**.01**
Aortic stenosis	6 (21)		20 (37)	.13
Root abscess	5 (17)		17 (31)	.44
Preoperative embolic events				
Brain/CNS	7 (24)		14 (26)	.89
Lungs	10 (35)		10 (19)	.11
Spleen	6 (21)		5 (9.3)	.26
Kidneys	5 (17)		5 (9.3)	.48
Eyes	0 (0)		1 (1.9)	1.0
Extremities	5 (17)		6 (11)	.66
Liver	0 (0)		2 (3.7)	.77
Heart	1 (3.4)		1 (1.9)	1.0
Other	2 (6.9)		5 (9.3)	1.0
Previous cardiac surgery				
CABG	0 (0)		2 (3.7)	.77
Aortic valve repair/replacement	4 (14)		8 (15)	1.0
Mitral valve repair/replacement	0 (0)		7 (13)	.11
Tricuspid valve repair/replacement	1 (3.4)		0 (0)	.75
Pulmonary valve repair/replacement	0 (0)		0 (0)	1.0
Ascending or arch repair/replacement	0 (0)		0 (0)	1.0
Incidence of cardiovascular surgery				
First surgery	24 (83)		39 (72)	.29
First reoperation	5 (17)		12 (22)	.59
Second or more reoperations	0 (0)		3 (5.6)	.50
Wait time, d	8 (4, 12)	6 (3, 13)		.54

Data presented as median (25%, 75%) for continuous data and n (%) for categorical data. The variables in bold are statistically significant. *CNS*, Central nervous system; *CABG*, coronary artery bypass graft.

∗ Not including valve valvotomy or valvuloplasty.

**Table E3:** Operative data in IV drug users

Variable	Survivor (n = 29)	Nonsurvivor (n = 54)	*P* value
Status			
Elective	2 (6.9)	0 (0)	.23
Urgent	26 (90)	44 (82)	.51
Emergent	1 (3.4)	10 (19)	.11
Circulatory arrest	1 (3.4)	2 (3.7)	1.0
CPB time, min	128 (82, 248)	154 (111, 228)	.32
Crossclamp time, min	100 (60, 184)	110 (80, 162)	.25
Aortic valve procedure			
Repair	0 (0)	0 (0)	1.0
Replacement	12 (41)	32 (59)	.12
Implantation technique∗			
Total	4 (14)	0 (0)	**.02**
Modified inclusion	4 (14)	11 (20)	.46
Mitral valve procedure			
Repair†	8 (28)	17 (32)	.71
Replacement	5 (17)	16 (30)	.22
Tricuspid valve procedure			
Repair	4 (14)	17 (32)	.08
Replacement	9 (31)	4 (7.4)	**.01**
Pulmonic valve procedure			
Repair	0 (0)	1 (1.9)	1.0
Replacement	0 (0)	0 (0)	1.0
Concomitant procedures			
CABG	1 (3.4)	5 (9.3)	.60
Aortic root procedure	8 (28)	15 (28)	.99
Ascending aorta procedure	1 (3.4)	2 (3.7)	1.0
Aortic hemiarch procedure	0 (0)	0 (0)	1.0
Total aortic arch procedure	0 (0)	0 (0)	1.0

Data presented as median (25%, 75%) for continuous data and n (%) for categorical data. The variables in bold are statistically significant. *CPB*, Cardiopulmonary bypass; *CABG*, coronary artery bypass graft.

∗ Implantation technique for stentless valves.

† Repair includes repair, reconstruction, or annuloplasty.

**Table E4:** Postoperative outcomes in IV drug users

Variable	Survivor (n = 29)	Nonsurvivor (n = 54)	*P* value
RBC units	1.0 (0.0, 3.0)	1.0 (0.0, 4.0)	.58
Ventilation hours	4.3 (1.9, 14)	12 (0.0, 37)	.31
ICU stay, d	2.2 (1.1, 4.1)	1.7 (0.0, 5.6)	.19
Reoperation for bleeding	0 (0)	2 (3.7)	.77
Planned delayed sternal closure	1 (3.4)	1 (1.9)	1.0
Sternal dehiscence	0 (0)	0 (0)	1.0
Sepsis	0 (0)	1 (1.9)	1.0
Positive blood cultures	1 (3.4)	1 (1.9)	1.0
Stroke	0 (0)	0 (0)	1.0
Paralysis	0 (0)	0 (0)	1.0
Prolonged ventilation	7 (24)	17 (32)	.48
Pneumonia	3 (10)	5 (9.3)	1.0
Device			
Pacemaker	5 (17)	5 (9.3)	.48
ICD	0 (0)	0 (0)	1.0
Cardiac arrest	0 (0)	1 (1.9)	1.0
Multisystem organ failure	0 (0)	0 (0)	1.0
Gastrointestinal event	0 (0)	2 (3.7)	.77
Atrial fibrillation	4 (14)	10 (19)	.81

Data presented as median (25%, 75%) for continuous data and n (%) for categorical data. *RBC*, Red blood cell; *ICU*, intensive care unit; *ICD*, implanted cardioverter defibrillator.

## Supplementary Data

Video 1 While IV drug use does not appear to affect perioperative mortality in patients with active infective endocarditis, it is a significant risk for long-term mortality. A multidisciplinary team is essential to mitigate this risk. Video available at: https://www.jtcvs.org/article/S2666-2736(22)00239-X/fulltext.
