# Supplementary figures and images for: Poor long-term outcomes of intravenous drug users with infectious endocarditis
Source: JTCVS Open. 2022 May 31;11:92–104. doi: 10.1016/j.xjon.2022.05.013 (PMC9510881; doi:10.1016/j.xjon.2022.05.013)

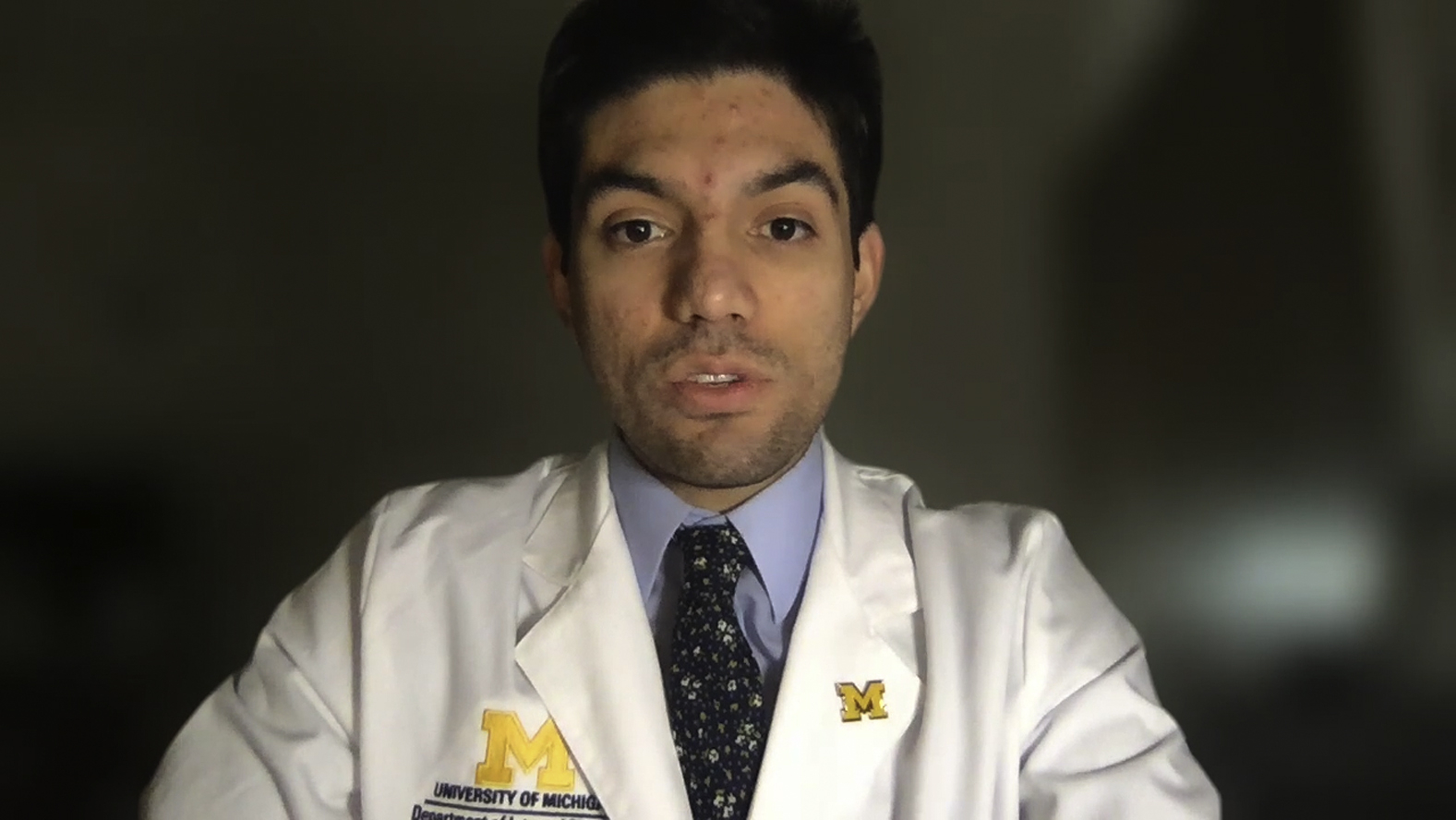

Supplement: Video 1 — While IV drug use does not appear to affect perioperative mortality in patients with active infective endocarditis, it is a significant risk for long-term mortality. A multidisciplinary team is essential to mitigate this risk. Video available at: https://www.jtcvs.org/article/S2666-2736(22)00239-X/fulltext. [file fx3.jpg]
